# Association Between Challenging Behaviour and Sleep Problems in Adults Enrolled in the Global Angelman Syndrome Registry

**DOI:** 10.1007/s10803-024-06367-6

**Published:** 2024-05-20

**Authors:** Heather Coleman, Arlene Mannion, Sally Whelan, Megan Tones, Helen Heussler, Matthew Bellgard, Geraldine Leader

**Affiliations:** 1 Irish Centre for Autism and Neurodevelopmental Research, School of Psychology, University of Galway, Galway, Ireland; 2 eResearch, Queensland University of Technology, Brisbane, Australia; 3 Children’s Health Queensland Hospital and Health Service, Brisbane, Australia; 4 Child Health Research Centre University of Queensland, Brisbane, Australia

**Keywords:** Angelman syndrome, Challenging behaviour, Global angelman syndrome registry, Adults, Sleep problems, Physical therapy

## Abstract

Angelman Syndrome (AS) is a rare genetic disorder that impacts 1:20,000 people. Challenging behaviour, such as severe injurious behaviour, aggression and frequent unprovoked episodes of laughter are a significant problem among adults with AS that adversely impacts an individual’s quality of life. This study, for the first time, aims understand the characteristic of challenging behaviour, its frequency, and the factors associated with it in adults with AS. Data from participants with AS (*N* = 37; aged 18–46 years) registered with the Global Angelman Registry, were divided into challenging behaviour and non-challenging behaviour groups based on the presence or absence of 50% of the behaviours recorded in the registry. Descriptive statistics, chi-squared and *t*-test analysis were conducted to assess the impact of variables on challenging behaviour. Multiple regressions were conducted to investigate the predictors of challenging behaviour. 56% of the sample presented with challenging behaviour. Disorders of arousal, self-injury, behaviour dysregulation, repetitive behaviour, and the lack of physical therapy accounted for 59% of the variance of challenging behaviour in this population. It was found that challenging behaviour was very common in this population. A significant association was found between challenging behaviour and both sleep arousal and the lack of physical therapy. Sleep arousal and the lack of physical therapy were the key factors associated with challenging behaviour in this study. Targeted interventions are needed to decrease challenging behaviour and future research should focus on sleep interventions and increased opportunities for physical therapy.

## Introduction

### Angelman Syndrome (AS)

Angelman Syndrome (AS) is a neurodevelopment disorder caused by deletion or abnormal expression of the UBE3A gene. AS is a rare genetic disorder with the current worldwide prevalence of AS estimated to range from 1/15,000 to 1/24,000 live births (Roche et al., 2020). It is associated with several physical, cognitive, and developmental abnormalities (Buiting et al., 2016; Heald et al., 2021). Typically, AS characteristics consist of an intellectual disability, ataxia, speech impairments, seizures, and patterns of unique behaviours consisting of a happy disposition and unprovoked episodes of laughter (Spruyt et al., 2018). Other symptoms include sleep disorders, feeding difficulties and in some cases, distinct facial features (Pearson et al., 2019).

Comorbidity refers to two or more medical conditions that coexist within an individual (Valderas., 2009). Hyperactive behaviour, anxiety and obsessive-compulsive pathology are psychiatric comorbidities in individuals with AS (Heald et al., 2020). Other common comorbid conditions in AS are epilepsy, scoliosis, gastrointestinal (GI) issues, and speech impairments (Besten et al., 2021). It has been estimated that 24–81% of individuals with AS also have a co-diagnosis of autism spectrum disorder (ASD) (Ellis et al., 2020; Moss et al., 2013; Leader et al., 2022b, c). This means that individuals with AS may also experience comorbidities that are common in ASD (Baker et al., 2018), such as speech impairments, epilepsy, anxiety, and GI issues (Gracio et al., 2018; Memari et al., 2012).

### Angelman Syndrome and Challenging Behaviour

Challenging behaviour are behaviours that threaten the quality of life of a person due to the intensity or frequency of the behaviour (McStay et al., 2013). They are also behaviours that can cause harm to the individual or others (Emerson, 1995; Watkins et al., 2019). Examples of common challenging behaviour in individuals with AS are pulling or grabbing people or hair, biting, pinching, and screaming. A particular characteristic of AS is unprovoked episodes of laughter and frequent smiling (Pearson et al., 2019). This unique behaviour has been reported across individuals of all ages with AS (Pearson et al., 2019; Williams et al., 2006). This behaviour can limit the persons social and community interactions which can in turn negatively impact the quality of life of the person. Another form of challenging behaviour seen in individuals with AS is self-injurious behaviour (SIB), such as head-banging (Leader et al., 2022b, c; Powis & Oliver, 2014; Watkins et al., 2019). Challenging behaviour in the form of aggression is reported in 6–10% of individuals with AS (Adams et al., 2011, 2020; Summers et al., 1995). In addition, behaviours that are considered benevolent in children may become more problematic and be classed as challenging behaviour when they are expressed in adults (Besten et al., 2021; Larson et al., 2014). Unprovoked episodes of laughter and smiling would be an example of this. It was also found that age significantly contributes to the frequency and severity of the variance in SIB (Leader et al., 2022b, c).

Challenging behaviours occur more often in the presence of social interaction compared to the absence of social interaction and adult attention may be a source of positive reinforcement for individuals with AS (Adams et al., 2020; Oliver et al., 2007). Challenging behaviour may also be maintained by access to adult attention, preferred items, or activities, and escape from the demand of less favourable tasks (Heald et al., 2021; Strachan et al., 2009).

It is also important to investigate the association of GI symptoms with challenging behaviour. GI symptoms including constipation, vomiting, difficulty swallowing, excessive swallowing and abnormal food related behaviours are common in individuals with AS (Glassman et al., 2017; Leader et al., 2022b, c) and are associated with challenging behaviour in individuals with limited communication skills (Adams., 2020; Buie et al., 2010; Glassman et al., 2017). Indeed, GI difficulties have been specifically linked with SIB (Wilde et al., 2017) and they may cause SIB (Richards et al., 2017).

Epilepsy in individuals with AS and its relationship to sleep disorders and challenging behaviours also requires further investigation. Epilepsy may also be linked in individuals with intellectual disability to SIB and to higher levels of aggression (Blickwedel et al., 2019; Deb & Hunter, 1991; Deb et al., 2020). Epilepsy affects the sleep and behaviour of individuals with AS (Besten et al., 2021; Thibert et al., 2013). It is reported that 20–80% of individuals with AS have a diminished need for sleep and abnormal sleep cycles (Hanzlik et al., 2020). Disorders of arousal are any behaviours arising while sleeping and range from confessional arousal which is abruptly waking in a confused state to night terrors (Loddo et al., 2019). These also include abnormal sleep-wake cycles, insomnia, sleep maintenance problems, and parasomnias. These result in reduced total sleep time, night awakenings, and nocturnal enuresis (Anderson et al., 2008; Sueri et al., 2017). Although sleep dysfunctions typically improve over an individual’s lifetime, they impact the life of adults with AS and their caregivers and they frequently require behavioural and pharmacological intervention (Hanzlik et al., 2020; Larson et al., 2014).

It is important to investigate the association of challenging behaviours with the history of communication development in individuals with AS, because, due to speech impairments, many individuals have difficulty requesting attention and maintaining interactions with others (Adams et al., 2020; Leader et al., 2022b, c; Strachan et al., 2009). Understanding developmental history is also important as many behaviours in individuals with AS change in frequency with age. For example, attention span increases with age (Adams et al., 2020; Clayton-Smith, 2001). However, speech impairments are common in AS at all ages (Pearson et al., 2019). In one study, 10–35% of individuals pinched, used aggression, and/or SIB to communicate with other people to reject, protest, and comment (Didden et al., 2009). Challenging behaviours may be maintained by the negative reinforcement that results from them (Didden et al., 2009; Heald et al., 2021). Functional communication training and speech therapy has been used effectively to treat challenging behaviour in individuals with AS (Gerow et al., 2018; Radstaake et al., 2012) and to encourage communication including nonverbal communication such as using pictures (van Buggenhout & Fryns, 2009; Wheeler et al., 2017). Other therapies may can also be required by individuals with AS and it can be hypothesised that reception of therapy may be associated with challenging behaviour. Individuals also can require physical therapy to help prevent deterioration of scoliosis, obesity, and because individuals may have an unstable gait or be unable to walk (Van der Burg et al., 2007; Wheeler et al., 2017). Occupational therapy may also be needed to improve and maintain fine motor and oral motor skills along with speech therapy as discussed above. However, further investigation is needed to better understand the factors that impact challenging behaviour in adults with AS. Greater understanding is needed to inform and improve therapies and interventions that aim to improve challenging behaviour.

The current study aimed to increase understanding of the factors that are associated with challenging behaviour in adults with AS, through examining how the factors predict their occurrence. It is important to further investigate challenging behaviour in AS because it’s presence can limit social and educational opportunities, community involvement, and result in the social exclusion of individuals with AS (Ellis et al., 2020). As described above, there is evidence that developmental history, and the presence of GI symptoms, epilepsy, sleep and communication problems are all associated with challenging behaviour (Leader et al., 2021; Leader at al., 2022; Bresciani et al., 2023). However, further investigation is now needed to better understand the nature and degree of the association between these factors and challenging behaviours in the context of AS. Improved understanding of these associated factors is necessary to facilitate the early identification of challenging behaviours and the factors that may exacerbate the behaviours or act as antecedents. To our knowledge examination of multiple associative factors in this context is novel. The findings of this research could usefully guide clinical decision-making and the administration and monitoring of therapeutic interventions that aim to improve challenging behaviour. The study used data gathered from the Global Angelman Syndrome Registry, which was created in 2016 (Tones et al., 2018; Napier et al., 2017). Firstly, data was examined to determine the frequency and the types of challenging behaviour. Having identified these behaviours, data from adults with AS who did or did not exhibit challenging behaviour were compared to examine their relationship to each of the following possible predictors of challenging behaviour: developmental history, GI symptoms, epilepsy, sleep problems, communication, and types of therapy the individual may have received.

## Method

### Sample

The study sample consisted of the data from 37 participants with AS, 45.9% of whom were male (*n* = 17) and 54.1% were female (*n* = 20). Participants ranged in age from 18 to 46.5 years with a mean age of 28.3 years (*SD* = 7.87). 54% (*n* = 20) of participants had chromosome deletion and 30% of participants (*n* = 11) were diagnosed with other non-deletion, while data was missing on the genetic basis of AS for 16% (*n* = 6) of participants.

### Procedure and Informants

Parents and caregivers of individuals with AS self-selected and registered with the Global Angelman Syndrome Registry website from their homes and provided their consent for data to be used. The inclusion criteria for the Angelman registry states that parents and caregivers are required to upload proof of Angelman Syndrome diagnosis when registering (Tones et al., 2018). Once registered parents and caregivers entered data through online questionnaires which are referred to as modules on the registry. The data for this study was accessed by researchers who contacted the Registry curator via the online form.

Within the first 12 months of the registry being initiated, 467 parents and caregivers registered individuals with a diagnosis of AS (Tones et al., 2018). Of the 467 individuals registered only 16% were adults (Tones et al., 2018). Currently, the registry has 2342 participants (The Global Angelman Syndrome Registry, n.d.).

The Global Angelman Syndrome Registry is advertised through many different means to recruit new participants. The Global Angelman Syndrome Registry attempts to recruit new participants through providing information about the registry at conferences that are attended by parents and caregivers of children with Angelman syndrome. They also advertise through the distribution of brochures and flyers about the registry to healthcare providers who work with Angelman syndrome families (The Global Angelman Syndrome Registry, n.d.). The registry is advertised on the GASR website as well as the GASR social media pages. Facebook has been recognised as successful way of advertising and engaging participants in registries (Gliklich et al., 2014; Tones et al., 2018) The Global Angelman Syndrome Registry is also widely advertised through its partnerships with different patient organisations all over the world. These partnerships have also led to the registry being translated into Chinese, French, Italian, and Spanish (Tones et al., 2018).

### Measures

#### Demographic Information

The sex of participants, current age, age of diagnosis, and type of AS were all found under Module 0 *Demographics* and Module 2 *History of Diagnosis and Results* in the Registry. The demographic details are summarised in Table 1.

**Table 1:** Summary of demographic information

Participant	Current Age	Sex	Type of Diagnosis	Presence of Challenging Behavior
1	23	Female	Other non deletion	Yes
2	24	Male	Chromosome deletion	No
3	27	Male	Other non deletion	Yes
4	46	Female	-	Yes
5	31	Female	Chromosome deletion	No
6	30	Male	Other non deletion	Yes
7	31	Male	Chromosome deletion	No
8	29	Female	Chromosome deletion	Yes
9	35	Female	Chromosome deletion	No
10	37	Female	Chromosome deletion	No
11	21	Male	Other non deletion	Yes
12	28	Female	-	Yes
13	15	Female	Chromosome deletion	Yes
14	18	Female	Other non deletion	Yes
15	19	Female	Other non deletion	Yes
16	21	Female	Chromosome deletion	Yes
17	29	Male	Chromosome deletion	No
18	27	Male	Chromosome deletion	Yes
19	25	Male	Other non deletion	No
20	28	Female	Other non deletion	Yes
21	18	Female	-	Yes
22	20	Male	Chromosome deletion	No
23	30	Female	Chromosome deletion	No
24	45	Female	Chromosome deletion	No
25	40	Male	Chromosome deletion	Yes
26	18	Male	Chromosome deletion	Yes
27	23	Female	-	Yes
28	38	Female	Chromosome deletion	Yes
29	23	Male	Other non deletion	No
30	32	Female	Chromosome deletion	Yes
31	38	Male	-	No
32	18	Female	Other non deletion	No
33	18	Male	Chromosome deletion	No
34	24	Male	Other non deletion	Yes
35	23	Male	Other non deletion	No
36	19	Male	-	No
37	39	Female	Chromosome deletion	Yes

#### Challenging Behaviour

Data on challenging behaviour was obtained through data in the Module 5, *Behaviour and Development* of the registry. The module contained 28 questions. Informants are asked to rate on a scale from one to ten how problematic they believed the individual’s behaviour to be. A rating of one signified no problems and a rating of ten signified major problems. An example of the challenging behaviours in question were repetitive movements, fear of strangers, frequent smiling at nothing in particular, aggressive behaviours such as biting, hair pulling, hitting, or grabbing and exhibiting self-harming.

#### Developmental History

Data on development history was retrieved from Module 1 *Newborn and infancy history*. Twenty questions in this module addressed difficulties maintaining or regulating proper body temperature, data on feeding during infancy, prior feeding habits such as breastfeeding, bottle feeding or feeding by another means. Data on the experience of feeding difficulties and assistance utilised if difficulties occurred was gathered alongside other breastfeeding related data. Questions were used to determine the individual’s temperament such as happy, placid, easy going, and affectionate in the first 12 months of their life. Data was gathered on health problems including any difficulties with suck/swallow, failure to gain weight, transitioning to solid food and if there were any reflux/gastro/oesophageal problems.

#### Gastrointestinal Symptoms

Data on GI symptoms were obtained from Module 3 *Illnesses or medical problems*, including whether the individual with AS had ever had, or was currently experiencing gastroesophageal reflux, constipation, and strep throat. If relevant, the age of the individual’s first diagnosis with the condition was noted, its severity, and whether medical treatment or surgical treatment was required, the nature of the treatment, and the age of the individual when the condition resolved. This module consisted of 12 questions. In most cases, questions were answered by choosing the most suited answer to the participant from a choice of five answers.

#### Epilepsy

Module 4 *Medical history* and Module 6 *Epilepsy* provided data on seizures. Data was gathered on the presence, control, and current status of seizures.

#### Language and Communication

Data was obtained from the communication module Module 4.5 *Communication* that had seventeen questions that addressed expressive language. Informants reported the individual’s best level of verbal language communication, the caregivers preferred method of communication with the individual with AS, and the latter’s ability to use communication methods or systems, including Pragmatic Organisation Dynamic Display (PODD) books (Porter and Cafiero, 2009), and Augmentative and Alternative Communication (AAC) (Ganz et al., 2011).

#### Medication and Therapy Received

Module 7 *Medications and Interventions* provided data on the medications, interventions and therapies that the individual with AS was currently being administered and why they were being used. The possible responses included a list of medication and the following therapies: physical, speech and language, occupational, hippo, hydro/aquatic, music, art, pet, behavioural or any other. Data was also gathered on the individual’s age when the therapy service started, the frequency of the therapy sessions, and the typical length of a therapy session.

#### Sleep Disturbance Scale for Children

The Sleep Disturbance Scale for Children (SDSC) (Bruni et al., 1996) consists of 26 items which are used to assess sleep-related behaviours in children and adolescents. The 26-item scale is scored using a one to five-point scale, one being never and five being always (daily). Bruni et al. (1996) have determined a cut off score of 39 which indicates sleep problems, and they conducted a psychometric evaluation of the SDSC that found an internal consistency ranging from 0.71 to 0.79, a test-retest reliability of 0.71 and a diagnostic accuracy of 0.91. SDSC has been used previously with adults with AS to evaluate clinical phenotypes of adults (Besten et al., 2021 to examine sleep disturbances (Bruni et al., 2004) and to determine the impact of seizure and gastroesophageal reflux on sleep and behaviour (Tones et al., 2019).

### Analyses

To determine who presented with challenging behaviour, the answers to the 30 behavioural questions were analysed. It was decided that parents who answered yes to 50% or more of the 30 questions indicated the presence of challenging behaviour. 50% indicated a high quantity of behaviours and allowed for equal division of the participants into two groups. If the parent answered no to less than 50% of the behavioural questions from the *Behavioural and Developmental* Module this indicated that challenging behaviour was not present for this individual for the purpose of the current analyses.

Data was analysed using IBM SPSS Statistics 26. Descriptive statistics were performed, and chi-square tests were used to assess the associations between challenging behaviour and the following nominal variables: developmental history, GI symptoms, communication/language, epilepsy, and types of therapy received. *T*-tests were used to assess the differences between challenging behaviour and sleep problems and to assess the difference between age of diagnosis and whether the individuals presented with challenging behaviour or not. A multiple linear regression was conducted on the variables below to further describe their relationship with and determine their effect on challenging behaviour. Variables were chosen for the multiple linear regression as they demonstrated significance in the chi-square and *t*-test analyses. Challenging behaviour was the dependent variable while physical therapy, disorders of arousal, self-injury, behaviour dysregulation, and repetitive behaviour were the independent variables.

## Results

### Challenging Behaviour

Data on challenging behaviour is presented in Table 2. 56% (*n* = 21) of the sample presented with challenging behaviour and 43% (*n* = 16) presented with no challenging behaviour according to the groups determined as described above. Missing data for this variable was present for five participants. The age of diagnosis for AS occurred at between one and 24 years of age. The age of diagnosis data was missing for 78% (*n* = 29) of participants. In the independent *t*-test conducted to compare age of diagnosis and those who presented with challenging behaviour (*M* = 6.00, *SD* = 10.16) and those who presented with no challenging behaviour (*M* = 1.64, *SD* = 0.55) there was not a significant difference reported. The magnitude of the means (means difference = 4.36, 95% CI [-10.47, 19.19]) was moderate (eta squared = 0.08). This means that the study found no statistically significant difference in the average age of diagnosis for people with and without challenging behaviour.

**Table 2:** Frequency and percentage of challenging behaviours

Behaviours	Presence of challenging behaviours			
	Yes		No	
	Frequency	Percentage	Frequency	Percentage
Appropriate Laughter	36	97.3	1	2.7%
Frequent Appropriate Smiling	35	94.6	1	2.7%
Good Concentration	34	91.9	1	2.7%
Poor Attention	33	89.2	3	8.1%
Will Socialise with Anyone	31	83.8	6	16.2%
Fascination with Water	29	78.4	8	21.6%
Oppositional Behaviours	28	75.7	8	21.6%
Separation Anxiety	26	70.3	10	27%
Anxious Behaviours	24	64.9	13	34.1%
Agitation in New Situations	23	62.2	14	37.8%
Grabbing	23	62.2	13	35.1%
Frequent Smiling at Nothing in Particular	23	62.2	14	37.8%
Fear of New Situations	20	54.1	17	45.9%
Spontaneous Laughter at Nothing in Particular	18	48.6	19	51.4%
Hyperactivity	18	48.6	19	51.4%
Hair Pulling	17	45.9	20	54.1%
Repetitive Focal Hand Movements	17	45.9	19	51.4%
Impulsivity	15	40.5	21	56.8%
Repetitive Slapping of the Wall	15	40.5	21	56.8%
Hitting	13	35.1	24	64.9%
Biting	12	32.4	25	67.6%
Fear of Being Left in School or in Care Situations	11	29.7	26	70.3%
Fear of Strangers	10	27	27	73%
Repetitive Whole-body Movements	10	27	24	64.9%
Night-time Laughter	9	24.3	28	75.7%
Self-harming Behaviour	9	24.3	28	75.7%
Head Banging	9	24.3	28	75.7%
Self-hitting	7	18.9	30	81.1%

### Challenging Behaviour and Developmental History

Chi-Square tests were performed examining the developmental history of the participants. The analysis was conducted with data missing for 22 of the participants. As presented in Table 3 below, no significant associations were found between the frequency of all challenging behaviours and the developmental history of participants.

**Table 3:** Summary of chi-square analyses for gastrointestinal symptoms and challenging behaviour

Variable	*n*	Challenging Behaviour			No Challenging Behaviour			Statistics
		%	Yes	No	%	Yes	No	
Breastfed During Infancy	33	54.5	9	9		8	6	0.41
Feeding Difficulties as a Newborn	33	54.5	12	6	45.5	12	3	0.46
Assistance Used in Feeding at Infancy	27	51.9	4	10	48.1	7	6	1.78
Refusal to Nurse	30	56.7	9	8	43.3	5	8	.62^3^
Could not Latch	29	58.6	7	10	41.4	7	5	.83^3^
Ineffective Suck	28	57.1	7	9	42.9	10	2	.05^2^
Biting During Breast Feeding	30	56.7	3	14	43.3	1	12	.61^2^
Vomiting	29	58.6	12	5	41.4	5	7	.15^2^
Arching	30	56.7	7	10	43.3	2	11	.23^2^
Excessive Movements	30	56.7	7	10	43.3	4	9	.71^2^
Irritability Associated with Feeding	30	56.7	7	10	43.3	5	2	.02^3^
Difficulties Transitioning to Solid Food	33	54.5	1	17	45.5	4	11	.15^2^
Happy in First 12 Months of Life	33	54.5	17	1	45.5	15	0	1.00^2^
Placid in First 12 Months of Life	33	54.5	11	7	45.5	8	7	.20^3^
Easy Going in First 12 Months of Life	33	54.5	13	5	45.5	14	1	.19^2^
Affectionate in First 12 Months of Life	32	53.1	14	3	46.9	12	3	1.00^2^
Difficulties with Suck/Swallow	32	53.1	14	3	46.9	11	4	.68^2^
Difficulties with Failure to Gain Weight	33	54.5	8	10	45.5	7	8	.02^3^
Reflux/Gastro/Oesophageal Problems	33	54.5	5	3	45.5	7	8	4.95^3^
Other Health Problems	31	54.8	10	7	45.2	8	6	.01^3^

^1^This figure was taken from Likelihood Ratio

^2^This figure was taken from Fisher’s Exact Test

^3^This figure was taken from the Chi-square analysis

### Challenging Behaviour and Gastrointestinal Symptoms

76% of those with constipation received treatment and 68% of individuals who experienced vomiting with feeds received medical treatment. 50% (*n* = 8) of individuals who were diagnosed with gastroesophageal reflux, experienced reflux at a severe level whilst 50% experienced it at a mild to moderate level. 64% (*n* = 21) of participants who experienced constipation experienced it at mild to moderate severity. Data was missing for two participants for this variable. 50% of participants (*n* = 10) experienced a one-off episode of strep throat per year. 79% (*n* = 15) of participants experienced strep throat at mild to moderate severity during episodes. Data was missing for 12 participants on this variable. Chi-Square tests were conducted to examine the association between GI symptoms and challenging behaviour of the participants. The results of the chi-square tests found no significant association between GI symptoms and challenging behaviour. The chi-square results are presented in Table 4.

**Table 4:** Summary of chi-square analyses for gastrointestinal symptoms and challenging behaviour

Variable	*n*	Challenging Behaviour			No Challenging Behaviour			Statistics
		%	Yes	No	%	Yes	No	
Individuals who experienced gastroesophageal reflux	33	51.5	4	13	48.5	7	9	1.52^3^
Medical treatment required	16	75	9	3	25	4	0	.53^2^
Surgical treatment required	17	70.6	2	10	29.4	0	5	1.00^2^
Individuals who experience constipation	35	54.2	14	5	45.8	15	1	.187^2^
Managed by medication	37	56.8	11	10	43.2	11	5	1.01^3^
Managed by diet	37	56.8	9	12	43.2	3	13	2.41^3^
Managed by other methods	37	56.8	19	2	43.2	12	4	.371^2^
Individuals who experienced vomiting with feeds	33	57.6	9	10	42.4	4	10	1.19^3^
Medical treatment required	21	61.9	6	7	38.1	1	7	.17^2^
Surgical treatment required	20	60	1	11	40	1	7	1.00^2^
Individuals who experience gagging	33	54.5	12	6	45.5	7	8	1.34^3^
Individuals who experienced strep throat	35	54.3	10	9	45.7	10	6	.35^3^

^1^This figure was taken from Likelihood Ratio

^2^This figure was taken from Fisher’s Exact Test

^3^This figure was taken from the Chi-square analysis

### Challenging Behaviours and Seizures

A Chi-Square test conducted to examine seizures in relation to challenging behaviour, indicated no significant association between challenging behaviour and seizures, χ^2^ (1, *n* = 35), *p* = .608. The expected count is less than five and therefore the *p* value was taken from Fisher’s Exact Test. Data was missing for 5% (*n* = 2) of participants for this variable. This suggests that there is no evidence to support that challenging behaviour is more common in participants who experienced seizures.

### Challenging Behaviour and Communication

Data was missing for 14 participants regarding communication. 35% (*n* = 10) of participants responded best to two step commands and 67% (*n* = 22) preferred communicating through gestures. 86% of participants used gestures to communicate, 65% of individuals used moans, and 62% communicated through signing. As shown in Table 5, no significant associations were found between communication variables and challenging behaviour using Chi-Square tests.

**Table 5:** Summary of chi-square analyses for communication and challenging behaviour

Variable				*n*	Challenging Behaviour			No Challenging Behaviour			Statistics
				%	Yes	No	%	Yes	No		
Uses Expressive Language				37	56.8	18	3	43.2	14	2	.55^1^
Uses Babbles				37	56.8	9	12	43.2	10	6	1.40^3^
Uses Moans				37	56.8	16	5	43.2	8	8	2.73^3^
Uses Single Words				37	56.8	6	15	43.2	6	10	.33^3^
Uses Intentional Sound				37	56.8	18	3	43.2	13	3	1.00^2^
Uses Two- or Three-Word Phrases				37	56.8	2	19	43.2	0	16	0.50 ^2^
Uses Longer Phrases				37	56.8	1	20	43.2	0	16	1.00^2^
Uses Spoken Word				36	58.3	7	14	41.7	4	11	.73^2^
Uses Gestures				36	58.3	19	2	41.7	12	3	.63^2^
Uses Signing				37	56.8	15	6	43.2	8	8	1.77^3^
Uses Visual Pictures				35	60	16	5	40	9	5	.47^2^
Uses Formal PODD Books				32	62.5	2	18	37.5	1	11	1.00^2^
Uses iPad Apps and Pictures to Voice				35	60	13	8	40	8	6	.08^3^
Uses Eye Tracking Devices				31	61.3	3	16	38.7	0	12	.27^2^
Uses Low Tech AAC				32	62.5	6	14	37.5	2	10	.68^2^
Uses Mid Tech AAC				31	61.3	5	14	38.7	3	9	1.00^2^
Uses High Tech AAC				33	57.6	9	10	42.4	8	6	.31^3^

^1^This figure was taken from Likelihood Ratio

^2^This figure was taken from Fisher’s Exact Test

^3^This figure was taken from the Chi-square analysis

### Challenging Behaviour and Therapy Received

A Chi-Square test indicated a significant association between challenging behaviour and the lack of physical therapy, χ^2^ (1, *n* = 37), *p* = .001. The expected count was less than five and therefore the *p* value was taken from Fisher’s Exact Test. Data on the therapy received by participants is displayed in Table 6 below. This reveals that there is a strong relationship between challenging behaviour and therapy received. The lack of physical therapy is likely to be a contributing factor to challenging behaviour.

**Table 6:** Summary of chi-square analyses for therapy received and challenging behaviour

Variables			*n*	Challenging Behaviour			No Challenging Behaviour			Statistics
				%	Yes	No	%	Yes	No	
Physical Therapy			37	56.8	1	20	43.2	8	8	0.00**^2^
Speech Therapy			37	56.8	2	19	43.2	6	10	.00^2^
Occupational Therapy			37	56.8	2	19	43.2	6	10	.06^2^
Hippotherapy			37	56.8	1	20	43.2	3	13	.30^2^
Hydrotherapy			37	56.8	3	18	43.2	4	12	.44^2^
Music therapy			37	56.8	4	17	43.2	5	11	.46^2^
Art Therapy			37	56.8	3	18	43.2	2	14	1.00^2^
Pet Therapy			37	56.8	0	21	43.2	0	16	-
Behavioural Therapy			37	56.8	0	21	43.2	1	15	.43^2^
Other Therapy			37	56.8	1	20	43.2	2	14	.57^2^

**p < .001

This figure was taken from Likelihood Ratio

This figure was taken from Fisher’s Exact Test

This figure was taken from the Chi-square analysis

### Challenging Behaviour and Sleep Problems

Twenty-one participants had missing data in the *SDSC* Module. All participants’ SDSC scores were below the cut-off point of 39 and ranged from a score of 7.11 to 12.48. The independent-samples *t*-test, conducted to compare the Sleep Disturbance Scale for Children (SDSC) subscale scores for participants with challenging behaviour and those without challenging behaviour. As presented in Table 7, there was a significant relationship between disorders of arousal and challenging behaviour. There was a significant difference in the independent *t*-test that compared disorder of arousal scores for those with challenging behaviour (*M* = 1.17, *SD* = 0.36) and those with no challenging behaviour (*M* = 1.00, *SD* = 0.00). The magnitude of the means (means difference = 0.17, 95% CI [-0.09, 0.42]) was moderate (eta squared = 0.10).

**Table 7:** Summary of t-test analyses for sleep problems and challenging behaviour

Variable	Challenging Behaviour				No Challenging Behaviour				*t*
	*n*	*%*	*M*	*SD*	*n*	*%*	*M*	*SD*	
Disorders of Initiating Sleep	11	50	2.16	0.56	11	50	2.06	0.88	0.33
Disorders of Sleep and Breathing	9	47.3	1.69	0.66	10	52.7	1.53	0.65	0.51
Disorders of Arousal	10	47.6	1.17	0.36	11	52.4	1.00	0.00	1.46*
Disorders of Sleep Wake Transitions	11	47.8	2.13	0.65	12	52.2	1.90	0.74	0.78
Excessive Somnolence	11	50	1.86	0.69	11	50	1.93	0.72	− 0.241
Sleep Hyperhydrosis	10	45.5	1.20	0.48	12	54.5	1.50	0.90	− 0.94

*p < .05

### Predictors of Challenging Behaviour

Results from the multiple linear regression are presented in Table 8. It found that the predictor variables of physical therapy, disorders of arousal, self-injury, behaviour dysregulation, and repetitive behaviour, accounted for 59% of the variance of challenging behaviour in this population. The results indicated negative beta values for the predictors disorders or arousal, self-injury, behaviour dysregulation and repetitive behaviour. These negative values indicate that as frequency of challenging behaviour increase these variables are showing a decrease in frequency. The beta values reveal that behaviour dysregulation (*β* = − 0.45, *p* < .05) was weighted most. Repetitive behaviour, (*β* = − 0.20, *p* = .36), lack of physical therapy (*β* = 0.22, *p* = .27), disorders of arousal (*β* = − 0.15, *p* = .40) and self-injury (*β* = − 0.07, *p* = .76) were not significant predictors in this model. There was a low risk of multicollinearity in the data.

**Table 8:** Summary of multiple linear regression model for predictors of challenging behaviour

Variable	β	F	ΔR²	Adj. ΔR²
Physical Therapy	0.22	4.35	0.59	0.46
Disorders of Arousal	− 0.15			.
Self Injury	− 0.07			
Behaviour Dysregulation	− 0.45*			
Repetitive Behaviour	− 0.20			

*p < .05

## Discussion

This study identified predictors of challenging behaviour in adults with AS. 58% of participants presented with challenging behaviour in the current sample. Challenging behaviour in the form of aggressive behaviours presented with higher rates in comparison with Summers et al. (1995) which reported, with a significantly smaller sample *n* = 11, that 10% of participants presented with challenging behaviour. A high frequency of smiling and episodes of unprovoked laughter were found in the current study. This frequency concurs with the findings of Oliver et al. (2007).

There were no significant findings observed to indicate that developmental history, GI symptoms, seizures or communication impacted challenging behaviour in participants. Further research is needed on early diagnosis and intervention as it was observed that participants who presented with challenging behaviour were diagnosed at a later age when compared to the mean age of diagnosis in participants who presented with no challenging behaviour. These findings are comparable to the findings of Deb and Hunter (1991) who reported that there was limited evidence that seizures were linked to higher levels of challenging behaviour in 150 participants. The findings of the current study also concur with those of Didden et al. (2009) and Heald et al. (2021) who reported that pre-linguistic form of communication and forms of gesturing signing were frequently used by individuals with AS.

The sleep problem of disorders of arousal had significant effects on challenging behaviour. Sueri et al. (2017) also reported high frequencies of sleep problems in individuals with AS, as this occurred with 90% of their sample. Adults with AS may have a diminished need for sleep (Sueri et al., 2017). However, this may impact parents and caregivers who are likely to suffer from sleep deprivation which can lead to fatigue, irritability, and feelings of helplessness. Adults with AS and their caregivers have been seen to benefit from behavioural and pharmacological intervention to help decrease sleep deprivation (Larson et al., 2014). Therefore, it is important that sleep problems are treated to decrease challenging behaviour in adults with AS.

A significant relationship between the lack of physical therapy and challenging behaviour was identified with 71% of participants who have never received physical therapy presented with challenging behaviour. There has been no previous literature on the association of physical therapy and challenging behaviour in individuals with AS. This strongly suggests the need for future research in the form of a randomized control trial (RCT) to investigate the effect of physical therapy on challenging behaviour.

In the current study, five predictors of challenging behaviour in adults with AS were identified. These were lack of physical therapy, disorders of arousal, self-injury, behaviour dysregulation, and repetitive behaviour. These predictors are important as they enable practitioners and clinicians to investigate underlying problems when presented with challenging behaviour in individuals. These findings may be informative to both physiotherapist and occupational therapists to promote the use of physical therapy to decrease challenging behaviour in adults with AS. These findings can inform psychologists of the importance of providing intervention for behaviour dysregulation before it possibly escalates to challenging behaviour. These findings may also be useful to parent’s and caregivers to be able to identify precursors of challenging behaviours such as behaviour dysregulation and ritualistic behaviour. This knowledge allows parents and care givers to intervene access supports before challenging behaviour occurs.

This research has limitations. Data was submitted to the registry by parents who self-selected and provided parental reports for their adult children. This may have introduced biases into the data (Tones et al., 2018) particularly due to language, literacy barriers, and inaccuracies due to incorrectly recalling information or errors in data entry (Gliklich et al., 2014) and because parents were required to recall specific information from many years previously. However, parent and care giver reports have been found to be reliable and consistent with clinical reports (Rydz et al., 2005). Gorrindo et al. (2012) in particular, found the results of evaluations conducted by pediatric gastroenterologists, were comparable with parental reports of GI symptoms in children with developmental disorders. Another limitation is the high level of missing data, especially in relation to participant’s developmental history and the study’s relatively small sample size.

Nevertheless, the study provides novel insight into the predictors of challenging behaviour in adults with AS and it is the first study to use the Global Angelman Syndrome Registry data to investigate challenging behaviour in this population. Future research will benefit from larger sample sizes as data, or participants with a greater diversity of demographic characteristics are added to this growing repository. Further research is needed to increase understanding of the relationship between challenging behaviour and epilepsy. Thibert et al. (2009) states that more than 80% of individuals with a diagnosis of AS also present with epilepsy. Although the current study found no statistical significance showing that epilepsy has an impact on challenging behaviour, previous studies have reported consist higher rates of behavior in epilepsy compared to non-epilepsy groups (Blickwedel et al., 2019; Deb & Hunter, 1991). In addition, research has shown that adults with AS have a diminished need for sleep and require full time care. Future research is needed on how disorders of arousal affect caregivers and how in turn this can affect adults with AS. The findings highlight that medication may impact challenging behaviour in adults with AS. It would be useful to investigate further the relationship between challenging behaviour and medication in adults with AS. Anti-epileptic drugs are used in controlling seizures in majority of individuals with AS (Besten et al., 2021). Therefore, we can assume that majority of adults with AS are using at least one form of medication. Finding more of these contributing variables such as medication may increase understanding of the predictors of challenging behaviour in adults with AS.

In conclusion, this study found that challenging behaviour was most commonly found in adults who experienced sleep problems or who did not receive physical therapy. There is a is a significant association between challenging behaviour, and behaviour dysregulation, sleep arousal and the lack of physical therapy in adults with AS. These findings can inform future research which is needed to evaluate suitable treatments and intervention to ameliorate challenging behaviour in adults with AS. These can target the predictors identified in this study, including sleep interventions and providing increased opportunities for physical therapy. These novel findings offer researchers and clinicians a greater insight into the predictors of challenging behaviour.
